# The contribution of vascular risk factors in neurodegenerative disorders: from mild cognitive impairment to Alzheimer’s disease

**DOI:** 10.1186/s13195-020-00658-7

**Published:** 2020-08-04

**Authors:** Yu-Wen Cheng, Ming-Jang Chiu, Ya-Fang Chen, Ting-Wen Cheng, Ya-Mei Lai, Ta-Fu Chen

**Affiliations:** 1 Department of Neurology, National Taiwan University Hospital, Hsin-Chu Branch, Hsin-Chu, Taiwan; 2 Graduate Institute of Clinical Medicine, College of Medicine, National Taiwan University, Taipei, Taiwan; 3 Department of Neurology, College of Medicine, National Taiwan University Hospital, National Taiwan University, No. 7 Chung-Shan South Road, Taipei, 10055 Taiwan; 4 Graduate Institute of Brain and Mind Sciences, College of Medicine, National Taiwan University, Taipei, Taiwan; 5 Graduate Institute of Psychology, National Taiwan University, Taipei, Taiwan; 6 Department of Medical Imaging, National Taiwan University Hospital, Taipei, Taiwan

**Keywords:** Alzheimer’s disease (AD), Low-density lipoprotein (LDL) cholesterol, Mild cognitive impairment (MCI), Plasma biomarkers, Vascular risk factors

## Abstract

**Background:**

Optimization of vascular risk factor control is emerging as an alternative approach to improve cognitive outcomes in Alzheimer’s disease, although its efficacy is still under debate. We aimed to investigate the contribution of vascular risk factors on Alzheimer’s biomarkers and conversion rate to dementia in subjects with mild cognitive impairment (MCI) with low cerebral small vessel disease burden.

**Methods:**

Two hundred ninety-five newly diagnosed MCI subjects were enrolled from March 2005 to May 2017 for a cross-sectional assessment of vascular risk factors and Alzheimer’s plasma and imaging biomarkers, followed by a cognitive outcome assessment 24 months after enrollment. The association between vascular risk factors and Alzheimer’s biomarkers were tested using multivariable linear regression models adjusted with age, gender, education, and APOE ε4 allele. The association between vascular risk factors and conversion to dementia was tested using multivariable logistic regression models adjusted with age, gender, education, and baseline Mini-Mental State Examination (MMSE) score.

**Results:**

At baseline, higher low-density lipoprotein (LDL) cholesterol level was associated with more advanced plasma biomarkers, including Aβ42/Aβ40 ratio (*P* = 0.012) and tau level (*P* = 0.001). A history of hypertension was associated with more advanced white matter hyperintensity (*P* = 0.011), while statin therapy for dyslipidemia was associated with less advanced white matter hyperintensity (*P* = 0.002). At 24 months, individual vascular risk factor was not significantly associated with cognitive outcome. By contrast, statin therapy for dyslipidemia was associated with reduced conversion to dementia (adjusted OR = 0.191, 95% CI = 0.062~0.586, *P* = 0.004).

**Conclusions:**

For MCI subjects, dyslipidemia may contribute to AD-related neurodegeneration while hypertension may contribute to vascular pathology. The association between statin therapy for dyslipidemia and reduced conversion to dementia supports further interventional study to evaluate the potential beneficial effect of statin in MCI subjects.

## Background

Alzheimer’s disease (AD) is the leading cause of dementia in the elderly population, and its rapidly increasing disease burden challenges health-care and socioeconomic systems worldwide [1]. Despite intense efforts in AD drug development in the past decades, there is so far no established disease-modifying therapy that can slow neurodegeneration in AD [2–4]. Traditional approaches for disease-modifying therapy target the amyloid cascade and tauopathy. By contrast, the optimization of vascular risk factor control is emerging as an alternative approach. Vascular cognitive impairment (VCI) refers to cognitive impairment associated with cerebrovascular disease [5]. While control of vascular risk factors remained the main treatment strategy for VCI, whether risk factor modification prevents cognitive decline remained controversial [6]. Approximately half of clinically probable AD subjects have mixed brain pathology, most commonly Alzheimer’s disease pathology and microvascular infarcts [7]. For patients with clinical AD, concurrent vascular pathology was associated with more advanced cognitive impairment in several autopsy [8, 9] and neuroimaging [10, 11] studies. These findings inspired the idea that therapies targeting vascular pathology may slow cognitive decline not only in patients with VCI but also in patients with clinical AD [12].

Observational studies support the idea that vascular risk factor control may reduce cognitive decline in the non-demented elderly population [13, 14] and in subjects with mild cognitive impairment (MCI) [15, 16]. The 2-year follow-up results of the Finnish Geriatric Intervention Study to Prevent Cognitive Impairment and Disability (FINGER) [17] showed significantly improved overall cognitive performance and executive function in at-risk elderly subjects who received multicomponent lifestyle intervention involving physical activity, nutritional guidance, cognitive training, social activities, and management of cardiovascular risk factors. Recently published results from the Systolic Blood Pressure Intervention Trial (SPRINT) showed that intensive blood pressure control (systolic blood pressure target < 120 mmHg) targeting hypertensive subjects with increased cardiovascular risk reduced the incidence of MCI [18]. However, the benefit of vascular intervention in subjects with preclinical AD or MCI has not yet been proven by randomized controlled trials [19]. The Evaluation of Vascular Care in Alzheimer’s Disease (EVA) study showed a non-significant result in either mesial temporal lobe atrophy or cognitive decline in subjects with clinical AD and concurrent cerebrovascular lesions who received multicomponent vascular care for 2 years [20, 21]. Additionally, whether vascular pathology acts independently or synergistically with Alzheimer’s pathology in causing cognitive decline remains under debate.

In this observational study, we aim to investigate the impact of vascular risk factors on Alzheimer’s disease-related pathology by (1) assessing the cross-sectional association between vascular risk factors and AD-specific plasma and imaging biomarkers and (2) assessing the association between baseline vascular risk factors and 2-year cognitive outcome in subjects with mild cognitive impairment (MCI) who have low concurrent cerebral small vessel disease (SVD) burden. Our hypothesis is that even in subjects with MCI and low cerebral SVD burden, vascular risk factors may not only contribute to cerebrovascular pathology, but also accelerate Alzheimer’s pathology. Through their contributions to both the cerebrovascular pathology and Alzheimer’s neurodegeneration, vascular risk factors may further accelerate cognitive decline.

## Methods

### Study design and participants

Subjects who visited the neurology clinic at National Taiwan University Hospital for cognitive complaints and were diagnosed to have MCI were recruited prospectively from March 2005 to May 2017. The age range for recruitment was 50 years or older. The diagnosis of MCI was made based on standardized neuropsychological assessment (Supplementary Table 1) and according to the core clinical criteria recommended by the 2011 National Institute on Aging-Alzheimer’s Association workgroups [22]. We excluded subjects who had major systemic diseases (e.g., *major* cardiopulmonary diseases, or advanced renal or hepatic diseases), neurological diseases (e.g., epilepsy, history of stroke, brain tumor, traumatic head injury, or other known neurodegenerative diseases suggested by medical history or neurological assessment), or psychiatric diseases that may interfere with cognitive performance; subjects who were illiterate or had significant visual or auditory problems that precluded them from neuropsychological assessment; and subjects who refused or were not able to receive brain MRI study. To limit our study population to MCI subjects with low cerebral SVD burden, we excluded subjects who had severe leukoaraiosis, defined as a Fazekas score of three, or lacunas on a brain MRI. After enrolment, participants received standardized vascular risk factor surveys, structural brain MRI exams, and plasma biomarker measurements. During the 2-year follow-up period, global cognitive performance and functional status were assessed using the Mini-Mental State Examination (MMSE) and Clinical Dementia Rating (CDR) scores at an annual interval or whenever a decline in cognitive function was reported by the participant or their family members. Conversion to clinical probable AD dementia was defined based on the core clinical criteria proposed by the 2011 National Institute on Aging-Alzheimer’s Association workgroups [23].

### Clinical information and vascular risk factors survey

Participants received a vascular risk factor survey with a structured questionnaire and a cross-sectional fasting blood test. The history of individual vascular risk factors, including hypertension, diabetes mellitus, dyslipidemia, and habitual tobacco smoking, was recorded. For each risk factor, disease status was coded as without relevant disease, with known disease and under medical treatment, or with known disease but not under medical therapy. Plasma biochemical measurements included total cholesterol, triglycerides, low-density lipoprotein (LDL) cholesterol, high-density lipoprotein (HDL) cholesterol, fasting plasma glucose, and hemoglobin A1c (HbA1c). Hypertension was defined according to the subject’s reported past history and verified by their medical record and active medication list. Subjects who reported having hypertension and either had documented hypertension in the medical record or were receiving antihypertensive agents were recorded as having hypertension. Dyslipidemia was defined whenever the subject had a total cholesterol level > 200 mg/dl, an LDL cholesterol level > 130 mg/dl (or > 100 mg/dl for subjects with concurrent diabetes mellitus), a triglyceride level > 200 mg/dl, or for whoever received a long-term lipid-lowering agent. Diabetes mellitus was defined according to the subject’s reported past history or if the patient had an HbA1c level > 6.5%. Habitual tobacco smoking was defined if the subject had smoked 100 or more cigarettes during their lifetimes.

Apolipoprotein E (*APOE*) genotyping was also performed to assess this possible confounding factor associated with Alzheimer’s biomarkers.

### Neuroimaging and plasma biomarkers

Brain magnetic resonance imaging (MRI) was performed on a 1.5 T MRI scanner. The scanning protocol included axial T2-weighted-fluid-attenuated inversion recovery (T2-FLAIR), fast spin-echo T2-weighted sequences, and a coronal T1-weighted image (T1WI) or a 3D T1WI. The axial slices were positioned to run parallel to a line that joins the most inferoanterior and inferoposterior parts of the corpus callosum and had a thickness of 5 mm with a gap of 1.5 mm. To represent AD-related early structural changes, the entorhinal cortical thickness and hippocampal volume were measured quantitatively on T1-weighted structural MRI scans using the FreeSurfer software (version 5.3) (http://surfer.nmr.mgh.harvard.edu/). The Desikan-Killiany cortical atlas was used for cortical parcellation [24]. White matter hyperintensity (WMH) was rated using a visual scoring system modified from Fazekas et al. [25], and each brain MRI scan was rated from 0 to 3 on the coronal view of the FLAIR series across the frontal and parietal lobes on either side. For each participant, we calculated the average WMH score from the abovementioned four brain areas, with a higher score indicating more extensive leukoaraiosis.

Subjects enrolled after July 2015 were included for plasma biomarker measurements. Plasma levels of amyloid β (Aβ)40, Aβ42, and total tau were detected using the immunomagnetic reduction (IMR) technique as published by Chiu et al. [26, 27]. As shown in previous studies using the IMR assay, an elevated plasma Aβ42/Aβ40 ratio and elevated plasma tau were detected in patients with AD [27, 28] and were associated with disease severity [27]. The plasma Aβ42 level detected by IMR was negatively associated with the CSF Aβ42 level determined by ELISA [29] and positively associated with cerebral amyloid deposits on PibPET [30]. The plasma tau level was negatively associated with memory functions and mesial temporal cortical thickness in subjects with MCI and AD [31].

Sample size was calculated using the software G*Power (version 3.1) to estimate the number of subjects needed to detect the relationship between vascular risk factors and plasma biomarkers in multiple linear regression models. To detect medium effects (effect size *f*^2^ = 0.15) of tested variables on plasma biomarker at a two-sided 0.05 significance level and under the correction of four covariates, a total of 92 subjects will be needed to achieve 80% power. Ninety-nine subjects were enrolled for plasma biomarker measurements.

### Statistical analysis

Independent *t* tests for continuous variables and chi-square tests or Fisher’s exact tests for categorical variables were used to compare the between-group differences in demographic variables. To assess the cross-sectional association between vascular risk factors and individual neurodegenerative biomarkers, we performed multivariable linear regression analysis. The association between individual vascular risk factors and neurodegenerative biomarkers were tested in linear regression models covariated with age, gender, education, and *APOE* genotype. We used the false discovery rate method proposed by Benjamini et al. to adjust for multiple comparisons [32]. Only vascular risk factors with the false discovery rate controlled below 0.05 were included for further stepwise regression analysis (Supplementary Table 2 and Supplementary Table 3). The forward stepwise selection method (*P* value < 0.1 for entry and *P* value > 0.2 for removal) was used to construct models of factors associated with AD biomarkers. Additional models covariated with history of lipid-lowering medications and plasma lipid measurements were constructed to assess the association between dyslipidemia and neurodegenerative biomarkers. To assess the association between vascular risk factors and cognitive outcome at 24 months, multivariable logistic regression models covariated with age, gender, education, and baseline MMSE score were applied to estimate the odds ratio (OR) and the 95% confidence interval (CI) for conversion to clinical probable AD dementia. A *P* value less than 0.05 was considered statistically significant. Data were analyzed using the statistical software PASW for Windows, version 22.0.

### Standard protocol approvals, registrations, and patient consents

The study was approved by the ethics committee of the National Taiwan University Hospital. All participants provided written informed consent before enrollment.

## Results

Two hundred and ninety-five MCI subjects were recruited from March 2005 to May 2017. The demographic profile of the participants is summarized in Table 1. The mean age of the participants was 72.7 years (SD 8.8). Fifty-six percent of the participants were female, 26% were *APOE* ε4 carriers, and the median educational level was 12.0 years (IQR 6.0–15.0). The median baseline MMSE score upon recruitment was 27.0 points (IQR 25.0–28.0). Eighty-three percent of the participants had amnestic-type MCI. Most of the participants with non-amnestic type MCI had impaired executive function. On the other hand, 46% of the participants with amnestic type MCI had concurrent executive function impairment.

**Table 1:** Demographics and biomarker profile of enrolled subjects

	Enrolled subjects (*N* = 295)
Age, years	72.7 ± 8.8
Gender, female %	56%
Educational level, years; median (IQR)	12.0 (6.0–15.0)
MMSE, median (IQR)	27.0 (25.0–28.0)
MCI type, %	
Amnestic MCI	83%
Non-amnestic MCI	17%
APOE4 (carrying one or more ε4 allele)	26%
Reported vascular risk factors (no history/under Tx/no Tx)^a^, %	
Hypertension, %	43/52/4%
Type 2 diabetes mellitus, %	83/16/1%
Dyslipidemia, %	67/20/13%
Smoking, (active smoker/quitted smoker), %	8/8%
Less than 1 pack per day	11%
1–2 packs per day	4%
More than 2 packs per day	1%
Serum lipid profile	
Total cholesterol, mg/dL	190.4 ± 38.0
LDL cholesterol, mg/dL	112.1 ± 32.9
HDL cholesterol, mg/dL	52.2 ± 14.7
Triglyceride, mg/dL	122.0 ± 68.7
HbA1c	6.09 ± 0.88
Fasting plasma glucose	102.0 ± 24.0
Statin use (% in dyslipidemia)	77 (41%)
Current BMI, kg/m^2^	23.7 ± 4.3
Imaging biomarkers	
Average Schelten’s score for MTA	1.62 ± 0.72
Average Fazekas score for WMH	0.90 ± 0.62
Entorhinal cortical thickness, mm	3.102 ± 0.429
Hippocampal volume, mm^3^	3283 ± 588.3

Data were represented as mean ± SD or percentage (%)

*Abbreviations*: *APOE* apolipoprotein E, *BMI* body mass index, *HDL* high-density lipoprotein, *LDL* low-density lipoprotein, *MMSE* Mini-Mental State Examination, *MCI* mild cognitive impairment, *MTA* mesial temporal atrophy, *WMH* white matter hyperintensity

^a^History of vascular risk factor profile was derived from a questionnaire and coded into three groups: no history (no history), positive history and with medication control (under Tx), and positive history and without medication control (no Tx)

Regarding concurrent vascular risk factors reported by the participants or their informants, 56% of the enrolled subjects had hypertension, 17% had diabetes mellitus, 33% had dyslipidemia, and 16% were habitual smokers. Most of the subjects who had hypertension or diabetes received regular medications for disease control. Among subjects who had dyslipidemia, only 41% of them received regular lipid-lowering medications (i.e., statin), 18% of them were aware of but did not receive medication for the disease, and 41% of them were not aware of their abnormal lipid profile before recruitment.

### Vascular risk factors and plasma biomarkers for Alzheimer’s disease

Ninety-nine subjects in the cohort had a plasma sample available for biomarker measurements. There was no significant difference in the prevalence of vascular risk factors between subjects with and without plasma biomarker measurements (Supplementary Table 4). Subjects who had a plasma sample available for biomarker analysis were younger (70.1 vs. 74.0 years old) and had a higher educational level (12.1 vs. 9.7 years) and a higher baseline MMSE score (27.1 vs. 26.0) than those who did not have an available plasma sample.

**Fig. 1:**
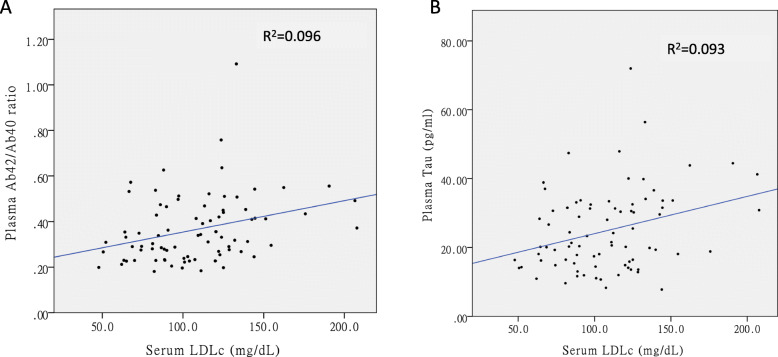
The association between LDL cholesterol level and plasma biomarkers. Serum LDL cholesterol level was positively associated with plasma Aβ42/Aβ40 ratio (**a**) and plasma tau (**b**). LDL, low-density lipoprotein; Aβ, amyloid beta

In the multiple linear regression model adjusted for age, gender, educational level, and *APOE* ε4 carrier status, the LDL cholesterol level was positively associated with the plasma tau level (B estimate = 0.146, 95% CI = 0.064~0.228, standardized β = 0.411, *P* value = 0.001). Both the LDL cholesterol level (B estimate = 0.001, 95% CI = 0.0003~0.002, standardized β = 0.297, *P* value = 0.012) and BMI (B estimate = 0.009, 95% CI = 0.002~0.017, standardized β = 0.276, *P* value = 0.017) were positively associated with the plasma Aβ42/Aβ40 ratio (Fig. 1). There was no significant association between a history of dyslipidemia and plasma biomarkers.

### Vascular risk factors and neuroimaging biomarkers for dementia

We applied a multiple linear regression model adjusted with age, gender, educational level, and *APOE* ε4 carrier status to test the association between vascular risk factors and imaging biomarkers, including hippocampal volume, entorhinal cortical thickness, and the severity of white matter hyperintensity. After adjustment for the abovementioned covariates, a history of dyslipidemia was associated with a larger hippocampal volume (B estimate = 167.05, 95% CI = 58.20~276.89, *P* value = 0.003) (Table 2, Model I-1). The association between a history of dyslipidemia and hippocampal volume remained significant after adjustment for lipid profiles and statin therapy (B estimate = 215.074, 95% CI = 79.738~350.411, *P* value = 0.002). To clarify the association between a history of dyslipidemia and hippocampal volume, participants were classified into four groups according to their history of dyslipidemia and lipid-lowering therapy status: subjects without dyslipidemia (group 1), subjects with known dyslipidemia and receiving statin therapy (group 2), subjects with known dyslipidemia and not under medication control (group 3), and subjects unaware of their dyslipidemia status before enrolment (group 4). Subjects with known dyslipidemia and not under medication control (dyslipidemia group 3) had a larger hippocampal volume than the other groups (B estimate = 90.55, 95% CI = 9.06~172.04, *P* value = 0.030) (Table 2, Model I-2). Regarding entorhinal cortical thickness, there was no statistically significant association between individual vascular risk factors and entorhinal cortical thickness after adjustment for multiple comparisons (Supplementary Table 2).

**Table 2:** Factors associated with imaging biomarkers of neurodegeneration related to Alzheimer’s disease

Dependent variable	Model	Independent variables	B estimate (95% CI)	Standardized β	*P* value^b^
Hippocampal volume	I-1	Age	− 23.01 (− 28.71~ − 17.30)	− 0.414	< 0.001**
		Gender	96.58 (− 9.99~203.15)	0.099	0.076
		Educational level	− 3.93 (− 15.21~7.35)	− 0.038	0.494
		APOEε4	− 174.78 (− 331.62~ − 17.93)	− 0.114	0.029*
		Dyslipidemia	167.05 (58.20~275.89)	0.159	0.003**
Hippocampal volume	I-2	Age	− 22.67 (− 28.45~ − 16.88)	− 0.408	< 0.001**
		Gender	89.25 (−17.90~196.40)	0.091	0.102
		Educational level	− 1.97 (− 13.24~9.29)	− 0.019	0.731
		APOEε4	− 158.91 (− 316.83~ − 1.00)	− 0.104	0.049*
		Dyslipidemia: Group 3^a^	90.55 (9.06~172.04)	0.116	0.030*
White matter hyperintensity	II	Age	0.024 (0.017~0.032)	0.345	< 0.001**
		Gender	− 0.129 (− 0.268~0.010)	− 0.104	0.068
		Educational level	− 0.004 (− 0.019~0.011)	− 0.030	0.601
		APOEε4	0.094 (−0.111~0.299)	0.048	0.4368
		Hypertension	0.174 (0.040~0.309)	0.139	0.011*
		Statin therapy	− 0.236 (− 0.386~ − 0.086)	− 0.167	0.002**

Age, gender, educational level, and APOEε4 carrier status were adjusted as covariates in all linear regression models. Models were built by stepwise selection method with *P* value of entry = 0.1 and stay = 0.2

*Abbreviations*: *APOE* apolipoprotein E, *LDL* low-density lipoprotein

^a^Dyslipidemia status was divided into four groups according to disease and treatment status: subjects without dyslipidemia (group 1), subjects with known dyslipidemia and received statin therapy (group 2), subjects with known dyslipidemia and not under medication control (group 3), and subjects unaware of their dyslipidemia status before enrollment (group 4)

^b^* = *P* < 0.05, ** = *P* < 0.01

Regarding white matter hyperintensity severity rated by the Fazekas score, hypertension was associated with more advanced white matter hyperintensity (B estimate = 0.174, 95% CI = 0.040~0.309, *P* value = 0.011), while statin therapy for dyslipidemia was associated with less advanced white matter hyperintensity (B estimate = − 0.236, 95% CI = − 0.386~ − 0.086, *P* value = 0.002; Table 2, Model II). To clarify the association between statin therapy and white matter hyperintensity, we controlled serum levels of total cholesterol, LDL cholesterol, HDL cholesterol, and triglyceride as possible covariates in the linear regression model. A history of statin therapy remained significantly associated with less advanced white matter hyperintensity (B estimate = − 0.230, 95% CI = − 0.383~ − 0.078, *P* value = 0.003), independent of the lipid profile.

We further tested the difference in white matter hyperintensity between subjects who had ever received statin therapy and subjects without statin therapy using analysis of covariance (ANCOVA), in the subgroup of subjects with dyslipidemia (*n* = 182). Subjects who received statin therapy had a 22% lower WMH score (95% CI = 0.063~0.449, *P* value = 0.01) after adjustment for age, gender, educational level, lipid profile, and hypertension.

### Vascular risk factors and cognitive outcome at 24 months

Two hundred eight participants completed a cognitive assessment at 24 months and were further included in the cognitive outcome analysis. Subjects who completed cognitive follow-up had a higher educational level (11.3 ± 4.5 years vs. 8.6 ± 4.7 years, *P* < 0.0005), a higher baseline MMSE score (26.6 ± 2.4 vs. 25.8 ± 3.3), and a thinner entorhinal cortical thickness (3.087 ± 0.452 mm vs. 3.234 ± 0.360 mm) (Supplementary Table 5) than those who were lost to follow-up within 24 months (*n* = 87).

Fifty-three participants (25%) converted to clinical probable AD dementia within 24 months. Among the non-converters, 25 subjects (12%) had improved cognitive performance, and 130 subjects (62.5%) had stable cognitive performance at 24 months. Univariate analysis showed that converters were older (76.7 ± 9.3 vs. 71.7 ± 8.2 years, *P* value < 0.0005), had a lower baseline MMSE score (27.0 ± 2.4 vs. 25.4 ± 2.2), more frequently had amnestic-type MCI (94% vs. 75%, *P* value = 0.006), and had more advanced imaging and plasma biomarker profiles for neurodegeneration (Supplementary Table 6) than non-converters. There was also a trend towards less frequent statin use (29% vs. 45%, *P* value = 0.076) for those who had dyslipidemia.

After adjustment for age, gender, educational level, and baseline MMSE score, there was no statistically significant association between individual vascular risk factor and conversion to clinical probable AD dementia at 24 months (Supplementary Table 3). By contrast, statin therapy for dyslipidemia was associated with a reduced risk of conversion to dementia (OR = 0.191, 95% CI = 0.062 ~ 0.586, *P* value = 0.004; Table 3, Model I). The association between statin therapy and reduced conversion to dementia remained significant after adjustment for a history of dyslipidemia (Table 3, Model II) or the serum lipid profile (Table 3, Model III). Under the regression model adjusted for age, gender, educational level, baseline MMSE, and statin therapy, there was a trend between serum LDL cholesterol level and conversion to dementia (OR = 1.012, 95% CI = 1.000~1.025, *P* value = 0.060; Table 3, Model IV).

**Table 3:** Factors associated with conversion to dementia within 24 months

Model	Variables	Adjusted odds ratio (95% CI)	*P* value^a^
I	Age	1.082 (1.026~1.141)	0.003**
	Gender	0.859 (0.342~2.157)	0.746
	Educational level	1.001 (0.901~1.112)	0.987
	MMSE	0.768 (0.631~0.934)	0.008**
	Statin therapy	0.191 (0.062~0.586)	0.004**
II	Age	1.062 (1.016~1.109)	0.008**
	Gender	0.610 (0.277~1.345)	0.221
	Educational level	1.094 (0.991~1.207)	0.074
	MMSE	0.760 (0.643~0.900)	0.001**
	Dyslipidemia	1.617 (0.718~3.644)	0.246
	Statin therapy	0.279 (0.099~0.788)	0.016*
III	Age	1.072 (1.019~1.128)	0.007**
	Gender	0.851 (0.322~2.247)	0.744
	Educational level	1.034 (0.928~1.153)	0.543
	MMSE	0.760 (0.625~0.924)	0.006**
	Total cholesterol	0.942 (0.865~1.025)	0.167
	LDL cholesterol	1.074 (0.985~1.171)	0.104
	HDL cholesterol	1.060 (0.965~1.164)	0225
	Triglyceride	1.010 (0.993~1.028)	0.252
	Statin therapy	0.274 (0.096~0.274)	0.016*
IV	Age	1.064 (1.015~1.116)	0.010*
	Gender	0.720 (0.303~1.712)	0.457
	Educational level	1.070 (0.966~1.185)	0.194
	MMSE	0.743 (0.618~0.894)	0.002**
	Statin therapy	0.320 (0.123~ −0.836)	0.020*
	LDL cholesterol	1.012 (1.000~1.025)	0.060

Age, gender, educational level, and baseline MMSE were adjusted as covariates in all logistic regression models. Models were built by stepwise selection method with *P* value of entry = 0.1 and stay = 0.2. The tested independent variables in Model I were vascular risk factors (hypertension, diabetes mellitus, dyslipidemia, and smoking) and statin use. Models II and III were adjusted for history of dyslipidemia and lipid profile, separately, to test the association between statin use and conversion to dementia. Model IV was adjusted for statin use, to test the association between lipid profile (total cholesterol, LDL cholesterol, HDL cholesterol, and triglyceride) and conversion to AD at 24 months

*Abbreviations*: *APOE* apolipoprotein E, *HDL* high-density lipoprotein, *LDL* low-density lipoprotein, *MMSE* Mini-Mental State Examination

^a^* = *P* < 0.05, ** = *P* < 0.01

## Discussion

**Fig. 2:**
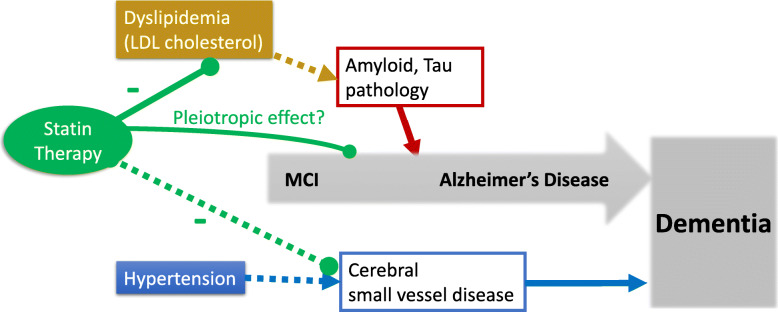
Proposed contribution of vascular risk factors to Alzheimer’s disease. For MCI subjects, dyslipidemia may contribute Alzheimer’s disease-specific neurodegeneration, and hypertension may contribute cerebrovascular pathology. Statin therapy may be beneficial for long-term cognitive outcome

In our cohort of MCI subjects with low cerebral SVD burden, the analysis of baseline biomarkers showed the following: (1) Higher LDL cholesterol was associated with plasma signatures of Alzheimer’s pathology. (2) Hypertension was associated with more advanced white matter hyperintensity, while statin therapy for dyslipidemia was associated with less advanced white matter hyperintensity. In the longitudinal analysis, statin therapy for dyslipidemia was associated with a reduced conversion rate to clinical probable AD dementia at 24 months. We confirmed the association between hypertension and white matter hyperintensity reported in several previous studies [33–35]. In addition, our findings suggest that dyslipidemia may contribute to Alzheimer’s disease-associated neurodegeneration and that statin therapy may contribute to better cognitive outcomes in MCI subjects (Fig. 2).

The association between midlife dyslipidemia and cerebral amyloid deposition was reported in several previous biomarker studies, especially focusing on non-demented elderly subjects [36, 37]. For late-life dyslipidemia, the association between lipid measurements and amyloid pathology was less consistent. Reed et al. reported a positive association between higher LDL cholesterol, lower HDL cholesterol, and the global PIB index in 74 subjects, including 33 cognitively normal and 38 MCI subjects [38]. However, Hughes et al. found no significant association between cholesterol measurements and cerebral Aβ deposition in 175 non-demented elderly subjects [39]. One important difference between these two studies is that Reed’s cohort was enriched for cerebrovascular disease and vascular risk factors. A key strength of our MCI cohort is that we enrolled subjects who had a low cerebral SVD burden on brain MRI. We still found consistent associations between elevated LDL cholesterol levels and plasma biomarkers for Alzheimer’s pathology, including the plasma Aβ42/Aβ40 ratio and plasma tau level.

Another important finding in our cohort is that statin therapy for dyslipidemia was associated not only with less advanced white matter changes at the time of MCI diagnosis but also with a reduced rate of conversion to dementia 24 months later. The above findings remained significant even if we controlled for lipid measurements as possible confounding factors. Several observational studies have suggested a potential beneficial effect of statin on cognitive outcome in non-demented elderly subjects [15, 40, 41]. However, randomized controlled trials failed to demonstrate any cognitive benefit of statin for either cognitively normal elderly individuals [42–44] or subjects with mild to moderate clinical probable AD [45–47]. Although randomized controlled trials are the gold standard for establishing causal relationships, they are often limited by small sample sizes and short follow-up periods. Additionally, subjects with abnormal lipid profiles were often excluded from previous randomized controlled trials. In the real world, dyslipidemia is a common disease in elderly individuals either with or without cognitive impairment. Subjects with concurrent dyslipidemia may be the target population that may especially benefit from statin therapy. The Simvastatin in Amnestic Mild Cognitive Impairment (MCI) Patients (SIMaMCI: clinicaltrials.gov NCT00842920) trial is an ongoing randomized placebo-controlled trial that tests the effect of simvastatin on the change in CDR at 24 months in subjects with amnestic-type MCI [48]. This trial includes MCI subjects with abnormal cholesterol measurements and may help clarify the cognitive effect of statin on MCI subjects with concurrent dyslipidemia.

We also found an unexpected association between a history of dyslipidemia and a larger hippocampal volume. Further analysis showed that it was those subjects who were aware of but did not receive medication for their dyslipidemia that contribute to the positive association with the hippocampal volume. One possible explanation is that those subjects who did not receive medication for dyslipidemia may take other means, such as lifestyle modification, to control their dyslipidemia. These lifestyle modifications may act as confounding factors that distort the association between dyslipidemia and neuroimaging measurements. The potential beneficial effects of physical activity [49, 50] and nutritional interventions [51, 52] on hippocampal volume in elder adults or MCI subjects were reported in several randomized controlled trials.

There were some limitations in this study. First, we did not have information for underlying AD pathology for the entire cohort. To restrict our study population to clinical MCI presumed to be due to Alzheimer’s pathology, we only included MCI subjects with low cerebral SVD burden on brain MRI. The concordance rate between clinical diagnosis of AD and Alzheimer’s pathology was 70–90% according to previous autopsy [7, 53] and amyloid PET [54] studies. Second, whether statin therapy exerts its beneficial effect on cognition through its lipid-lowering effect or other pleotropic effects cannot be fully answered by this study. We tried to address this issue by adjusting for lipid measurements in regression models and still found a significant association between statin therapy and better cognitive outcome. However, the long-term control status of dyslipidemia may not be reflected by this single-time-point-lipid measurement. In addition, we cannot exclude the possibility that, instead of being a causative factor, statin therapy may be a surrogate marker that reflects concurrent lifestyle modification or self-health awareness in the participants. Another limitation of this study is the potential recall errors associated with the vascular risk factor questionnaire. However, because the questionnaire was recorded upon the diagnosis of MCI and before conversion to dementia, it is unlikely that the recall error will bias the results towards a specific direction.

## Conclusions

In conclusion, our findings suggest that dyslipidemia and hypertension contribute to different neurodegenerative processes in MCI. Dyslipidemia, especially a higher LDL cholesterol level, may participate in AD specific neurodegeneration while hypertension may contribute to cerebrovascular pathology. Furthermore, statin therapy may play a role in slowing the conversion from MCI to dementia. Further studies are needed to elucidate the mechanism underlying the interaction between vascular pathology and Alzheimer’s pathology. The potential beneficial effect of statin therapy for dyslipidemia at or before the stage of MCI should be verified by randomized controlled trials. Understanding the role of vascular pathology in neurodegeneration may provide an alternative therapeutic approach for AD.

## Supplementary information

**Additional file 1.**

## Data Availability

Anonymized datasets analyzed during the current study are available from the corresponding author on reasonable request.

## Abbreviations

AD: Alzheimer’s disease
Aβ: Amyloid β
APOE: Apolipoprotein E
CDR: Clinical Dementia Rating
CI: Confidence interval
HbA1c: Hemoglobin A1c
HDL: High-density lipoprotein
IMR: Immunomagnetic reduction
IQR: Interquartile range
LDL: Low-density lipoprotein
MCI: Mild cognitive impairment
MMSE: Mini-Mental State Examination
MRI: Magnetic resonance imaging
OR: Odds ratio
SD: Standard deviation
SVD: Small vessel disease
T1WI: T1-weighted image
T2-FLAIR: T2-weighted-fluid-attenuated inversion recovery
WMH: White matter hyperintensity
