# Supplementary material for: The contribution of vascular risk factors in neurodegenerative disorders: from mild cognitive impairment to Alzheimer’s disease
Source: Alzheimers Res Ther. 2020 Aug 4;12:91. doi: 10.1186/s13195-020-00658-7 (PMC7401210; doi:10.1186/s13195-020-00658-7)
Supplement: Supplementary file 1 — Additional file 1. [file 13195_2020_658_MOESM1_ESM.docx]

**Supplemental Materials**

**Supplementary Table 1. Neuropsychological assessment batteries used for the diagnosis of MCI**

| Cognitive Domains | Neuropsychiatric assessment batteries |
| --- | --- |
| Global cognitive function | Mini-Mental State Examination (MMSE)  Clinical Dementia Rating (CDR) |
| Episodic memory | Benton Visual Retention Test (BVRT)  Logical memory subtests of the Wechsler Memory Scale Version III  Word sequence learning test |
| Executive function, working memory | Digit Span forward and backward  Trail making test  Semantic verbal fluency test  Wisconsin Card Sorting test |
| Visuospatial function | 3D block construction |
| Language function | Visual naming  Token test |

**Supplementary Table 2. Association between individual vascular risk factors and plasma and imaging biomarkers**

| Biomarker | Vascular Risk Factors | B estimate (SE)^a^ | Standardized β^a^ | P-value ^a^ | Benjamini-Hochberg p-value^b^ |
| --- | --- | --- | --- | --- | --- |
| Plasma tau | Hypertension | -2.474 (2.70) | -0.096 | 0.356 | 0.607 |
|  | Diabetes Mellitus | 3.209 (3.52) | 0.097 | 0.364 | 0.607 |
|  | Dyslipidemia | -0.409 (2.72) | -0.016 | 0.881 | 0.881 |
|  | Smoking | 2.396 (4.14) | 0.073 | 0.565 | 0.709 |
|  | BMI | 0.528 (0.38) | 0.170 | 0.172 | 0.430 |
|  | Statin therapy | -1.751 (3.05) | -0.061 | 0.567 | 0.709 |
|  | Total cholesterol, mg/dL | 0.107 (0.04) | 0.348 | 0.0046** | 0.023* |
|  | LDL cholesterol, mg/dL | 0.146 (0.04) | 0.411 | 0.00065** | 0.0065** |
|  | HDL cholesterol, mg/dL | 0.044 (0.10) | 0.054 | 0.652 | 0.724 |
|  | Triglyceride, mg/dL | -0.032 (0.02) | -0.172 | 0.132 | 0.430 |
| Plasma Aβ42/Aβ40 ratio | Hypertension | -0.017 (0.03) | -0.059 | 0.564 | 0.851 |
|  | Diabetes Mellitus | -0.016 (0.039) | -0.041 | 0.687 | 0.859 |
|  | Dyslipidemia | 0.017 (0.029) | 0.060 | 0.558 | 0.851 |
|  | Smoking | 0.036 (0.046) | 0.091 | 0.441 | 0.851 |
|  | BMI | 0.010 (0.004) | 0.300 | 0.013* | 0.043* |
|  | Statin therapy | -0.001 (0.033) | -0.003 | 0.977 | 0.977 |
|  | Total cholesterol, mg/dL | 0.0011 (0.0004) | 0.259 | 0.0099** | 0.043* |
|  | LDL cholesterol, mg/dL | 0.00049 (0.00017) | 0.173 | 0.0036** | 0.036* |
|  | HDL cholesterol, mg/dL | 0.00012 (0.0011) | 0.013 | 0.916 | 0.977 |
|  | Triglyceride, mg/dL | -0.00012 (0.00023) | -0.059 | 0.596 | 0.851 |
| Hippocampal volume | Hypertension | 5.179 (52.90) | 0.005 | 0.922 | 0.922 |
|  | Diabetes Mellitus | 29.168 (69.04) | 0.022 | 0.673 | 0.781 |
|  | Dyslipidemia | 167.05 (55.30) | 0.159 | 0.00275** | 0.025* |
|  | Smoking | 48.40 (79.23) | 0.035 | 0.542 | 0.781 |
|  | Statin therapy | 43.227 (58.94) | 0.039 | 0.464 | 0.781 |
|  | Total cholesterol, mg/dL | -1.074 (0.764) | -0.078 | 0.161 | 0.362 |
|  | LDL cholesterol, mg/dL | -1.596 (0.888) | -0.097 | 0.0734 | 0.220 |
|  | HDL cholesterol, mg/dL | -0.819 (2.079) | -0.022 | 0.694 | 0.781 |
|  | Triglyceride, mg/dL | 0.729 (0.405) | 0.095 | 0.0726 | 0.220 |
| Entorhinal Cortical thickness | Hypertension | 0.012 (0.038) | 0.017 | 0.761 | 0.856 |
|  | Diabetes Mellitus | -0.018 (0.050) | -0.020 | 0.725 | 0.856 |
|  | Dyslipidemia | 0.029 (0.038) | 0.043 | 0.450 | 0.810 |
|  | Smoking | 0.046 (0.057) | 0.049 | 0.423 | 0.810 |
|  | Statin therapy | -0.002 (0.043) | -0.003 | 0.963 | 0.963 |
|  | Total cholesterol, mg/dL | -0.001(0.001) | -0.063 | 0.280 | 0.810 |
|  | LDL cholesterol, mg/dL | -0.001(0.001) | -0.113 | 0.049* | 0.221 |
|  | HDL cholesterol, mg/dL | 0.00047(0.002) | -0.018 | 0.753 | 0.856 |
|  | Triglyceride, mg/dL | 0.001 (0.0003) | 0.113 | 0.042* | 0.221 |
| White matter hyperintensity | Hypertension | 0.161 (0.069) | 0.128 | 0.021* | 0.095 |
|  | Diabetes Mellitus | 0.025(0.091) | 0.015 | 0.785 | 0.785 |
|  | Dyslipidemia | -0.072 (0.069) | -0.058 | 0.297 | 0.535 |
|  | Smoking | -0.115 (0.104) | -0.066 | 0.270 | 0.535 |
|  | Statin therapy | -0.223 (0.077) | -0.158 | 0.0039** | 0.035* |
|  | Total cholesterol, mg/dL | 0.001 (0.001) | 0.029 | 0.611 | 0.785 |
|  | LDL cholesterol, mg/dL | 0.00045 (0.001) | 0.022 | 0.702 | 0.785 |
|  | HDL cholesterol, mg/dL | -0.003(0.003) | -0.063 | 0.271 | 0.535 |
|  | Triglyceride, mg/dL | 0.00033(0.001) | 0.033 | 0.545 | 0.785 |

^a^ The B estimates, standardized βs, and p-values were derived from linear regression models adjusted for age, gender, education, and *APOE* ε4 carrier status.

^b^ Multiple comparison was corrected by false discovery rate (FDR)

**Supplementary Table 3. Association between individual vascular risk factors and conversion to dementia at 24 months**

| Vascular Risk Factors | Adjusted OR (95% CI)^a^ | P-values | Benjamini-Hochberg P-value^b^ |
| --- | --- | --- | --- |
| Hypertension | 0.620 (0.275~1.400) | 0.250 | 0.336 |
| Diabetes Mellitus | 6.159 (1.245~30.459) | 0.026* | 0.117 |
| Dyslipidemia | 0.519 (0.223~1.205) | 0.127 | 0.286 |
| Smoking | 2.076 (0.581~7.412) | 0.261 | 0.336 |
| Statin therapy | 0.191 (0.062~ 0.586) | 0.004** | 0.034* |
| Total cholesterol, mg/dL | 1.005 (0.994~1.016) | 0.370 | 0.416 |
| LDL cholesterol, mg/dL | 1.010 (0.998~ 1.023) | 0.109 | 0.286 |
| HDL cholesterol, mg/dL | 1.004 (0.976~ 1.033) | 0.758 | 0.758 |
| Triglyceride, mg/dL | 0.996 (0.988~ 1.003) | 0.237 | 0.336 |

^a^ Adjusted OR were derived from logistic regression models adjusted for age, gender, education, and MMSE

^b^ Multiple comparison was corrected by false discovery rate (FDR)

**Supplementary Table 4. Demographics and biomarker profile of Enrolled Subjects**

|  | Subjects enrolled for plasma biomarkers ^a^ (N=99) | Subjects without available plasma sample (N=196) | p-value ^b^ |
| --- | --- | --- | --- |
| Age, years | 70.1 ± 9.1 | 74.0 ± 8.3 | <0.0005** |
| Gender , female % | 57% | 55% | 0.811 |
| Educational level, years | 12.1 ± 4.1 | 9.7 ± 4.8 | <0.0005** |
| MMSE | 27.1 ± 2.2 | 26.0 ± 2.9 | <0.0005** |
| MCI type, % |  |  | 0.632 |
| Amnestic MCI | 85% | 83% |  |
| Non-amnestic MCI | 15% | 17% |  |
| APOE4 (carrying one or moreε4 allele) | 22% | 33% | 0.154 |
| Reported Vascular Risk factors ^c^, % |  |  |  |
| Hypertension, % | 52/ 45/ 3 % | 39/ 56/ 5 % | 0.112 |
| Type 2 Diabetes Mellitus, % | 82/ 18/ 0 % | 84/ 15/ 1 % | 0.481 |
| Dyslipidemia, % | 62/ 26/ 12 % | 70/ 18/ 13 % | 0.268 |
| Smoking, % | 19% | 15% | 0.312 |
| Serum Lipid profile |  |  |  |
| Total cholesterol, mg/dL | 184.4 ± 37.9 | 193.4 ± 37.8 | 0.079 |
| LDL cholesterol, mg/dL | 107.7 ± 33.6 | 114.4 ± 32.4 | 0.140 |
| HDL cholesterol, mg/dL | 52.5 ± 14.6 | 52.0 ± 14.8 | 0.796 |
| Triglyceride, mg/dL | 116.2 ± 62.6 | 124.9 ± 71.5 | 0.344 |
| Statin use (% in dyslipidemia) | 27 (48 %) | 50 (39 %) | 0.408 |
| Current BMI | 23.5 ± 4.0 | 23.8 ± 4.4 | 0.681 |
| Imaging Biomarkers |  |  |  |
| Average Schelten’s score for MTA | 1.51 ± 0.71 | 1.67 ± 0.72 | 0.058 |
| Average Fazekas score for WMH | 0.71 ± 0.56 | 0.99 ± 0.63 | <0.0005** |
| Entorhinal cortical thickness, mm | 3.157 ± 0.435 | 3.094 ± 0.433 | 0.399 |
| Hippocampal volume, mm3 | 3474 ± 665.0 | 3280 ± 585.8 | 0.046* |
| Plasma Biomarkers, N=99 |  |  |  |
| Plasma tau, pg/ml | 25.83 ± 12.84 | NA | NA |
| Plasma Aβ42, pg/ml | 17.07 ± 3.31 | NA | NA |
| Plasma Aβ40, pg/ml | 50.93 ± 11.74 | NA | NA |

Data were represented as mean ± SD or percentage (%). Aβ=amyloid beta, APOE=Apolipoprotein E, BMI= body mass index, HDL= high-density lipoprotein, LDL=low-density lipoprotein, MMSE= Mini-Mental State Examination, MCI= mild cognitive impairment, MTA= mesial temporal atrophy, WMH= white matter hyperintensity.

^a^ Plasma biomarker measurements were available for subjects enrolled after July 2015.

^b^ Independent T-test and Chi-square test were used to compare demographic variables between subjects with and without plasma available for biomarker analysis. *=p<0.05, **= p<0.01.

^c^ History of vascular risk factor profile were derived from questionnaire and coded into three groups: no history, positive history and with medication control, and positive history and without medication control

**Supplementary Table 5. Comparison of demographic profile between subjects regularly followed for 24 months and those who lost follow up within 24 months.**

|  | Followed for >=24 months  (n=208) | Lost to follow-up before 24 months (n=87) | P-value ^a^ |
| --- | --- | --- | --- |
| Age, years | 73.0 ± 8.8 | 72.0 ± 8.8 | 0.405 |
| Gender , female % | 54 % | 59 % | 0.499 |
| Educational level, years | 11.3 ± 4.5 | 8.6 ± 4.7 | <0.0005** |
| MMSE | 26.6 ± 2.4 | 25.8 ± 3.3 | 0.027* |
| Amnestic-type MCI, % | 82 % | 87 % | 0.236 |
| APOE4, N= 114 vs 40 | 29 % | 14% | 0.097 |
| Vascular Risk factors ^b^, % |  |  |  |
| Hypertension, % | 46/ 50/ 4 % | 38 / 58/ 5 % | 0.419 |
| Type 2 Diabetes Mellitus, % | 84/ 15/ 1 % | 81/ 19/ 0 % | 0.504 |
| Dyslipidemia, % | 70/ 19/ 11 % | 61/ 23/ 17 % | 0.259 |
| Smoking, % | 18 % | 12 % | 0.278 |
| Serum Lipid profile |  |  |  |
| Total cholesterol, mg/dL | 193.2 ± 39.7 | 183.2 ± 32.4 | 0.058 |
| LDL cholesterol, mg/dL | 114.8 ± 34.2 | 105.6 ± 28.5 | 0.050 |
| HDL cholesterol, mg/dL | 52.0 ± 14.6 | 52.6 ± 15.0 | 0.792 |
| Triglyceride, mg/dL | 121.1 ± 72.5 | 124.3 ± 58.6 | 0.739 |
| AC glucose | 101.4 ± 22.5 | 102.7 ± 29.0 | 0.694 |
| HbA1c | 6.08 ± 0.90 | 6.04 ± 0.78 | 0.838 |
| Statin use (% in HLP, N=182) | 37 (48%) | 8 (29%) | 0.074 |
| Current BMI | 23.7 ± 4.27 | 23.8 ± 4.24 | 0.911 |
| Imaging characteristics |  |  |  |
| Average Schelten’s score for MTA | 1.64 ± 0.73 | 1.55 ± 0.68 | 0.337 |
| Average Fazekas score for WMH | 0.91 ± 0.60 | 0.86 ± 0.66 | 0.512 |
| Entorhinal cortical thickness, mm | 3.087 ± 0.452 | 3.234 ± 0.360 | 0.026* |
| Hippocampal volume, mm3 | 3329.3 ± 653.5 | 3498.8 ± 566.8 | 0.110 |
| Plasma biomarkers | (N=79) | (N=20) |  |
| Plasma tau, pg/ml | 26.72 ± 13.57 | 22.33 ± 8.93 | 0.173 |
| Plasma Aβ42, pg/ml | 17.25 ± 3.47 | 16.34 ± 2.51 | 0.274 |
| Plasma Aβ40, pg/ml | 49.89 ± 11.12 | 55.05 ± 13.43 | 0.079 |
| Plasma Aβ42/ Aβ40 ratio | 0.374 ± 0.151 | 0.317 ± 0.103 | 0.117 |

Data were represented as mean ± SD or percentage (%). Aβ=amyloid beta, APOE=Apolipoprotein E, BMI= body mass index, HDL= high-density lipoprotein, LDL=low-density lipoprotein, MMSE= Mini-Mental State Examination, MCI= mild cognitive impairment, MTA= mesial temporal atrophy, WMH= white matter hyperintensity.

^a^ Independent T-test and Chi-square test were used to compare demographic variables between subjects with and without plasma available for biomarker analysis. *=p<0.05, **= p<0.01.

^b^ History of vascular risk factor profile were derived from questionnaire and coded into three groups: no history, positive history and with medication control, and positive history and without medication control

**Supplementary Table 6. Univariate comparison between converters and non-converters**

|  | Non-Converter(n=155) | Converter (n=53) | P-value ^a^ |
| --- | --- | --- | --- |
| Age, years | 71.7 ± 8.2 | 76.7 ± 9.3 | <0.0005** |
| Gender, female % | 52 % | 62 % | 0.179 |
| Educational level, years | 11.4 ± 4.5 | 11.1 ± 4.5 | 0.721 |
| MMSE | 27.0 ± 2.4 | 25.4 ± 2.2 | <0.0005** |
| Amnestic-type MCI, % | 77 % | 94% | 0.006** |
| APOE4 (carrying at least one ε4 allele), | 28 % | 31% | 0.762 |
| Vascular Risk factors ^b^, % |  |  |  |
| Hypertension, % | 48/ 48/ 4 % | 40 / 56/ 4 % | 0.620 |
| Type 2 Diabetes Mellitus, % | 82/ 17/ 1 % | 90/ 10/ 0 % | 0.317 |
| Dyslipidemia, % | 66/ 23/ 11 % | 82/ 8/ 10 % | 0.046* |
| Smoking, % | 20 % | 12% | 0.226 |
| Serum Lipid profile |  |  |  |
| Total cholesterol, mg/dL | 191.8 ± 40.7 | 197.2 ± 37.1 | 0.417 |
| LDL cholesterol, mg/dL | 112.3 ± 35.2 | 121.4 ± 30.7 | 0.120 |
| HDL cholesterol, mg/dL | 52.0 ± 14.3 | 51.6 ± 16.1 | 0.956 |
| Triglyceride, mg/dL | 123.2 ± 79.4 | 115.4 ± 49.5 | 0.523 |
| AC glucose | 102.4 ± 24.4 | 98.4 ± 15.6 | 0.287 |
| HbA1c | 6.07 ± 0.92 | 6.10 ± 0.87 | 0.903 |
| Statin use (% in HLP, N=133) | 44 (45%) | 10 (29%) | 0.076 |
| Current BMI | 24.0 ± 4.6 | 22.7 ± 2.8 | 0.100 |
| Imaging characteristics | (N=98) | (N=26) |  |
| Average Schelten’s score for MTA | 1.56 ± 0.73 | 1.90 ± 0.66 | 0.002** |
| Average Fazekas score for WMH | 0.83 ± 0.58 | 1.15 ± 0.61 | 0.001** |
| Entorhinal cortical thickness, mm | 3.149 ± 0.442 | 2.851 ± 0.415 | 0.002** |
| Hippocampal volume, mm3 | 3412.7 ± 659.6 | 3014.9 ± 532.2 | 0.005** |
| Plasma biomarkers | (N=69) | (N=10) |  |
| Plasma tau, pg/ml | 25.06 ± 13.28 | 38.16 ± 9.74 | 0.004** |
| Plasma Aβ42, pg/ml | 16.94 ± 3.49 | 19.38 ± 2.53 | 0.037* |
| Plasma Aβ40, pg/ml | 50.33 ± 10.70 | 46.85 ± 13.95 | 0.358 |
| Plasma Aβ42/ Aβ40 ratio | 0.363 ± 0.152 | 0.446 ± 0.132 | 0.019* |

Data were represented as mean ± SD or percentage (%).BMI= body mass index, HDL= high-density lipoprotein, LDL=low-density lipoprotein, MMSE= Mini-Mental State Examination, MCI= mild cognitive impairment, MTA= mesial temporal atrophy, WMH= white matter hyperintensity.

^a^ Independent T-test and Chi-square test were used to compare demographic variables between subjects with and without plasma available for biomarker analysis. *=p<0.05, **= p<0.01.

^b^ History of vascular risk factor profile were derived from questionnaire and coded into three groups: no history, positive history and with medication control, and positive history and without medication control
